# Microstructure Evolution of Cement Paste Based on a Continuous Hydration Model for Irregular Polyhedral Particles

**DOI:** 10.3390/ma18112414

**Published:** 2025-05-22

**Authors:** Hong Huang, Zhigang Zhu, Yichen Li, Huisu Chen

**Affiliations:** 1 Faculty of Civil Engineering and Mechanics, Jiangsu University, Zhenjiang 212013, China; 2212223009@stmail.ujs.edu.cn (H.H.); 15637268801@163.com (Y.L.); 2 State Key Laboratory of Engineering Materials for Major Infrastructure, Jiangsu Key Laboratory of Construction Materials, School of Materials Science and Engineering, Southeast University, Nanjing 211189, China; chenhs@seu.edu.cn

**Keywords:** cement hydration, irregular particles, numerical simulation, degree of hydration

## Abstract

Hydration is a critical process in cement-based materials. The microstructure and macroscopic properties of cementitious composites are determined by a number of intricate chemical interactions. Previous studies on cement hydration have primarily focused on spherical and regular polyhedral particles. However, cement particles exhibit asymmetrical geometries. Therefore, this study develops a method to construct irregular polyhedral particles. A continuum-based hydration model of non-spherical cement particles (HYD-NSP), from our previous study, was employed to predict the evolution of microstructure of cement paste with irregular polyhedral particles. The results revealed that irregular polyhedral particles, with higher specific surface areas than spherical or regular polyhedral ones, significantly enhanced the degree of hydration and reduce the porosity in cement paste.

## 1. Introduction

Cement serves as a binding material that is extensively utilized in the fields of construction and civil engineering [1,2]. The process during which cement gradually transforms from a fresh state to a hardened state is referred to as cement hydration. Essentially, cement hydration involves the hardening of the cement paste to form a diverse range of unique microstructures. The hydration process of cement is an incredibly complex chemical reaction. It significantly influences the macroscopic properties of cement, such as mechanical and transport properties. Therefore, understanding cement hydration mechanisms and microstructural evolution can not only enhance concrete durability and mechanical performance but also promote the development of multi-scale simulation methods. These insights further enable high-performance cementitious composites, driving sustainable development in intelligent construction.

In the past decades, many researchers have utilized computers to simulate the microstructural evolution of cement paste. They have proposed a variety of hydration models, which can be mainly classified into two types: discrete-based models and continuous-based models.

In discrete modeling, Bentz and Garboczi developed the first model, CEMHYD3D [3,4], to simulate cement hydration based on digital image-based techniques. First of all, the system space and spherical cement particles were discretized into voxel points of the same size and dimensions. Subsequently, the digital cement particles were segmented into phases through thresholding and image processing. Finally, the dissolution, diffusion, nucleation, and reaction processes of voxel points were controlled using the cellular automata technique. Bullard et al. [5] employed the Virtual Cement and Concrete Testing Laboratory (VCCTL) to simulate various cement shapes and track the effects of these hydration microstructures on the hydration kinetics and setting of Portland cement. Additionally, they compared the effects of particle shape on the degree of hydration and solid-phase connectivity at different water–cement ratios (*w*/*c*). It was observed that particle shape affected the hydration of the cement paste in the early stage, mainly because of the specific surface area and the permeability lattice formed by the hydration products. Liu et al. [6] developed a new center-growth model and particle-filling algorithm to simulate the hydration process of cement with different shapes using the CEMHYD3D model. The results revealed that the effect of particle shape on hydration was significant and became less pronounced as the *w*/*c* ratio decreased. It is worth noting that the discretized model needs to handle a large number of discrete units. With the increase of simulation scale and accuracy requirement, the computational volume will grow exponentially, demanding a large number of computational resources and time. Therefore, these models exhibit significant resolution-dependent limitation during cement hydration.

In contrast, continuum-based hydration models rely on mathematical morphology to construct particle shape models. These models are no longer limited by computer resolution, and they can obtain simulation results relatively quickly, making them suitable for large-scale engineering simulation and rapid prediction. The main continuum-based models are the HYMOSTRUC [7,8], IPKM [9,10], etc. In the early stage, continuum-based hydration models were limited by the computational power of computers, so that they usually simplified the cement particles into spherical particles, which dramatically reduced the calculation burden but also differs from cement particle shape. Then, Zhu et al. [11] developed a new hydration model for non-spherical cement particles based on the HYMOSTRUC model. They employed Platonic particles to depict the shape of cement particles, demonstrating the hydration kinetics and the formation of hydration products of non-spherical cement particles. Moreover, they explored the evolutions of the microstructure within the hydrated cement paste. Results revealed that the influence of the shape of cement particles on the degree of hydration and the porosity of cement paste was mainly reflected by their specific surface area. Guo et al. [12] employed X-ray computed tomography (XCT) to obtain 3D images of cement particles and simulate the hydration process and the resultant microstructural evolution of cement paste. It was revealed that the *w*/*c* ratio and temperature significantly affected the pore structure and connectivity. Numerous studies have demonstrated that changes in particle morphology unavoidably result in modifications to the microstructure, which then impact macroscopic characteristics. Currently, the hydration models primarily use regular particles (such as spherical [7,8], ellipsoidal [13], and Platonic particles [11]) as the research objects; there are no publications on the hydration simulation of irregular particles. The morphology of irregular cement particles is relatively similar to that of real cement particles. Therefore, it is extremely necessary to carry out the research on irregular particles.

In this paper, we utilize the hydration kinetic equation to explore the evolution of the hydration microstructure based on the hydration reaction of irregular icosahedral particles, under HYD-NSP, the abbreviation for continuous-based hydration model of non-spherical cement particles. This exploration includes analyzing the trend of the degree of hydration and porosity in the representative volume element (RVE) of cement. Moreover, we analyze how these trends correlate with the shape of cement particle, *w*/*c* ratio, and the curing temperature.

## 2. Microstructure of Particle Packing in Cement Paste

In terms of chemical composition, Portland cement is mainly composed of four minerals after high-temperature calcination, namely alite (C_3_S), belite (C_2_S), aluminate (C_3_A), and a ferrite phase (C_4_AF). The hydration reaction of cement simply means that when cement contacts water, various chemical components in the cement and water undergo a series of chemical reactions so that the cement paste is gradually transformed from a plastic paste state to a rigid solid state. The most important part of the hydration products is hydrated calcium silicate (C-S-H gel) [14,15], mainly produced by the hydration of C_3_S and C_2_S, accounting for about 50~70% of the total hydration products [16]. It has good gelling and bonding properties and is the primary source of strength and durability of cement stones. In the non-spherical cement hydration model (HYD-NSP), the dense C-S-H gel corresponds to the inner products of cement hydration according to the density of the hydration products. Loose C-S-H gel corresponds to the external products of cement hydration. Both types of hydration products are distributed around the surface of unhydrated cement particles.

### 2.1. Construction of an Individual Cement Particle

**Figure 1:**
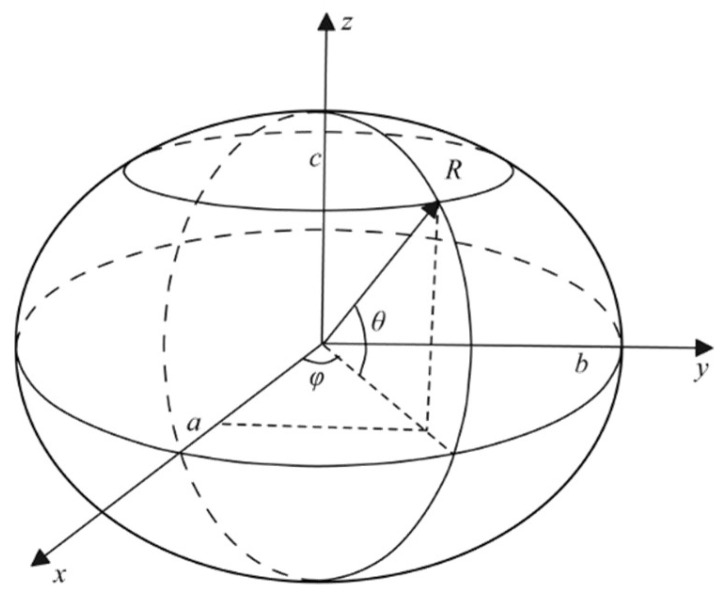
Ellipsoidal surface in a three-dimensional coordinate system.

Theoretically, considering the irregularity of the faces on the surface of cement particles, the cement particles can be represented by irregular polyhedral particles with spherical triangular dissections [17,18]. In this study, points were selected on the surface of ellipsoids and triangulated to accurately simulate natural particle morphology while accounting for their geometric irregularities. When a simple convex polyhedron is generated by triangulation, its outer surfaces are made up of triangular faces. Each edge on these triangles is shared by two faces. Combining this with Euler’s formula [19], the relationship is given by Equation (1).

(1) $$\left\{\begin{array}{l} v-e+f=2\\ 3f=2e \end{array}\right.$$

where *v*, *e*, and *f* represent the number of vertices, edges, and faces of the polyhedron. Therefore, the relationship between the number of faces *f* and the number of vertices *v* of a simple convex polyhedron can be derived, namely *v* = 1/2 × *f* + 2. Based on this, irregular polyhedral particles with any even number of faces can be constructed. To make the construction process of a polyhedron more intuitive, an irregular hexahedron was taken as an example. Initially, five points were randomly selected on the surface of the ellipsoid as vertices of the polyhedron. The shape of the ellipsoid in the 3D coordinate system is shown in Figure 1.

The coordinates of a point *P*(*x*, *y*, *z*) on the surface of ellipsoid can be expressed by Equation (2).

(2) $$\left\{\begin{array}{l} x=R\cos\theta \cos\varphi ,\;\theta \in [\frac{-\pi }{2},\frac{\pi }{2}]\\ y=R\cos\theta \sin\varphi ,\;\varphi \in \left[0,2\pi \right]\\ z=R\sin\theta \end{array}\right.$$

where *a*, *b*, and *c* represent the semi-axis lengths of the ellipsoid along the *x*-axis, *y*-axis, and *z*-axis, respectively. *R* is the distance from the origin to any point on the ellipsoid surface. The first spatial angle *θ* is defined as the angle between the vector (from the center of the ellipsoid to a point on its surface) and its projection in the *oxy* plane, while the second spatial angle *φ* is the angle between the projection of this vector in the *oxy* plane and the positive direction of the *x*-axis.

**Figure 2:**
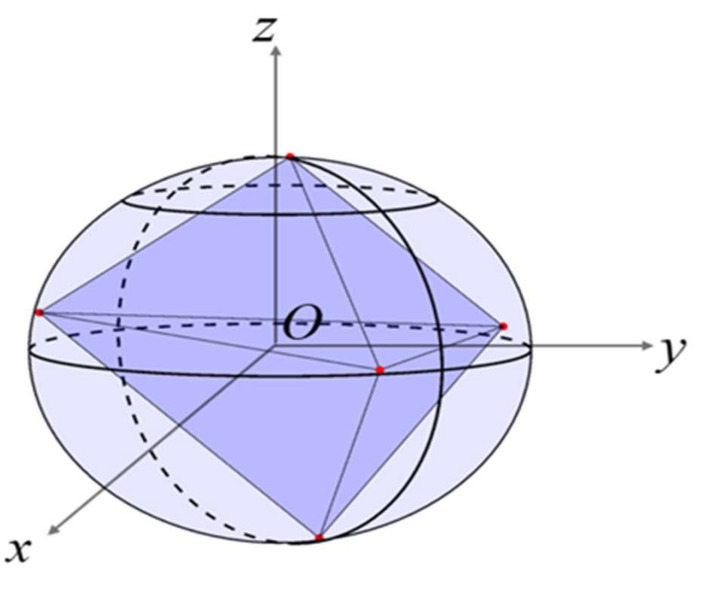
The construction of an irregular hexahedral particle in an ellipsoid.

These five points were then triangulated to generate hexahedral particles with irregular geometric shapes. This section introduces a key indicator *s* for measuring the shape characteristics of the polyhedron to facilitate the subsequent calculations. *s* is a sphericity, which is defined as the ratio of the surface area of a sphere with equivalent volume to the object to the actual surface area of the object. It can be given by Equation (3).

(3) $$s=\frac{\pi^{\frac{1}{3}}\times {\left(6V\right)}^{\frac{2}{3}}}{A}$$

where *A* is the surface area, and *V* is the volume of the particle. Based on the above method, numerous irregular hexahedral particles can be constructed. Take an irregular hexahedral particle as an example, as shown in Figure 2. Furthermore, these structures of other polyhedra with non-triangular faces can also be obtained by triangulating these non-triangular faces.

**Figure 3:**
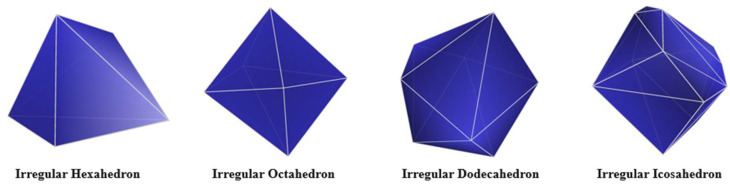
Four types of irregular polyhedral particles.

Therefore, any irregular polyhedron with an even number of faces (≥4 faces) can be constructed. In this study, an irregular hexahedral particle, an irregular octahedral particle, an irregular dodecahedral particle, and an irregular icosahedral particle were taken as examples, as shown in Figure 3.

### 2.2. Fresh State of Cement Paste

The microstructure of a fresh cement paste represents an unstable suspension system, where cement particles constitute the solid phase and water serves as dispersion medium. In the fresh state, the particle size distribution pattern of cement particles usually satisfies the Rosin–Rammler distribution [7,20,21,22], as given in Equation (4).

(4) $$G(D_{eq})=1-exp({-bD}_{eq}^{n})$$

where *G*(*D_eq_*) is the cumulative volume probability; *D_eq_* is the equivalent diameter of the cement particles; which is defined as the diameter of a sphere having the same volume as the irregular cement particles; and *b* and *n* are the two constants. The volume-based probability density function can be calculated by Equation (5).

(5) $$g(D_{eq})=\frac{dG(D_{eq})}{\left(dD_{eq}\right)}=b\times n\times D_{eq}^{n-1}\times exp(-bD_{eq}^{n})$$

Considering the particle size distribution to satisfy the Rosin–Rammler function, the particle size distribution was divided into several intervals, as shown in Table 1.

Theoretically, *M* in Equation (6) can be chosen at will; however, the number of small-sized particles in the cement powder is absolutely dominant [23]. Thus, in this paper, *M* was set to 50. The equivalent diameter of the *i*-th particle in the interval is given in Equation (6).

(6) $$D_{eq,i}=(2^{5i/M}-1)\frac{D_{eq\_max}-D_{eq\_min}}{2^{5}-1}+D_{eq\_min},0\leq i\leq M$$

In simulation, particles within a specific size range are typically placed into a given cubic container, which serves as a (RVE) for hydration simulations. The actual values of the cement fineness determine the minimum *D_eq_min_* and maximum *D_eq_max_* particle sizes of the cement particles in this context. The volume of cement in the range of particle sizes (*D_eq_* − *dD*, *D_eq_*) can be calculated by Equation (7).

(7) $$V_{\leq D_{eq}}=\frac{m_{c}}{\rho_{c}}\times G\left(D_{eq}\right)$$

The corresponding number of cement particles in this interval is given in Equation (8).

(8) $$N(D_{eq})=\frac{(m_{c}/\rho_{c})\times g(D_{eq})\times dD}{(\pi D_{eq}^{3}/6)\times 10^{-12}}=\frac{m_{c}\times b\times n\times D_{eq}^{n-1}\times exp(-bD_{eq}^{n})dD}{\rho_{c}\times (\pi D_{eq}^{3}/6)\times 10^{-12}}$$

where *m_c_* and *ρ_c_* are the mass and density of cement, respectively.

The volume fraction *ϕ_c_* of cement particles can be derived from Equation (9), based on a given *w*/*c* ratio (which is defined as the ratio of the mass of water *m_w_* to the mass of cement *m_c_*).

(9) $$\phi_{c}=\frac{m_{c}/\rho_{c}}{m_{c}/\rho_{c}+m_{w}/\rho_{w}}=\frac{m_{c}/\rho_{c}}{m_{c}/\rho_{c}+m_{c}\times w_{c}/\rho_{w}}=\frac{1}{1+w_{c}\times \rho_{c}/\rho_{w}}$$

where *m_w_* and *ρ_w_* are the mass and density of water. In this study, *ρ_c_* = 3.15 g/cm^3^, *ρ_w_* = 1 g/cm^3^.

To account for the influence of multiple factors on the hydration rate of the cement particle structure, this paper adopts the concept of spatial computational units from the HYMOSTRUC model [7]. A spatial computational unit refers to a series of small and relatively independent regions obtained by spatially dividing a cement paste. Each such unit can be regarded as a microscopic “reactor” that contains specific amounts of components like cement particles, water, hydration products, and pores. It is assumed that the physicochemical properties, including temperature, water content, and degree of cement hydration, are homogeneous within each unit.

## 3. Simulation of Hydration Process and Microstructure of Cement Pastes

In the hydration simulation, the shape of cement is represented by irregular icosahedral particles. This section describes the construction method of inner products, outer products, and unhydrated cores of an individual irregular polyhedral cement particle. Moreover, considering the particle size distribution of the particles in the cement paste, the degree of hydration and porosity of cement paste were calculated under the simulated multi-scale cement particles.

### 3.1. Hydration Mechanism of an Individual Irregular Polyhedral Particles

**Figure 4:**
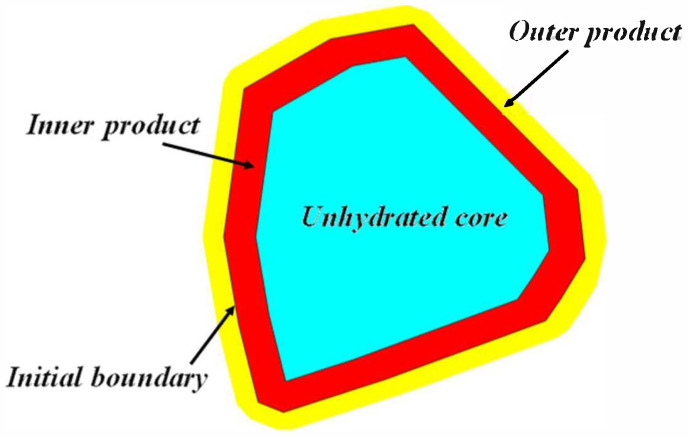
Schematic diagram of the hydration of an individual irregular polyhedral cement particle.

For an individual irregular polyhedral cement particle, the hydration reaction process can be regarded as an equal-thickness inward shrinkage that generates an inner product layer and an outward expansion that forms an outer product layer along the direction normal to each surface of the initial polyhedron. The volume of cement particles participating in the reaction is determined by the penetration depth, which is equivalent to the thickness of the inner products layer (the distance from the original surface of the particle to the new surface of the unhydrated part of the particle), and the shape of the remaining unhydrated part. Theoretically, the shape of the remaining unhydrated part is also a polyhedral structure that evolves from the initial irregular polyhedron. From a geometric perspective, the inner and the outer product layers can be envisioned as spheres of different sizes moving on the inner and outer surfaces of the original cement particles, respectively. This is similar to the Minkowski addition theory [11], where the diameters of the spheres are equivalent to the thicknesses of the inner and outer products layer, respectively, as shown in Figure 4. By means of this understanding, the geometric form and changing law of hydration products during the hydration process of cement can be more intuitively comprehended and analyzed.

Regarding this type of convex polyhedron structure, its volume can be obtained by summing up the volumes of several small triangular pyramids. For individual cement particle, the hydration rate *α_Deq_*_,*j*_ is the ratio of the volume of the hydrated portion to the initial volume, which is given by Equation (10).

(10) $$\alpha_{D_{eq},j}=\frac{V_{D_{eq},j}}{V_{D_{eq}}}$$

**Figure 5:**
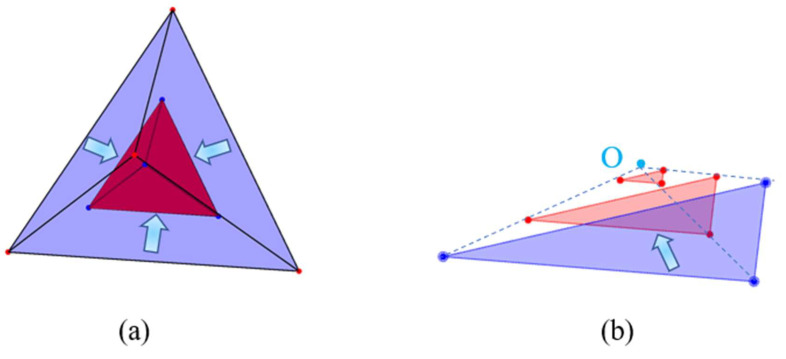
Schematic diagram of (**a**) the equal thickness shrinkage of a tetrahedron, and (**b**) the equal thickness shrinkage of an individual triangular surface at one point.

**Figure 6:**
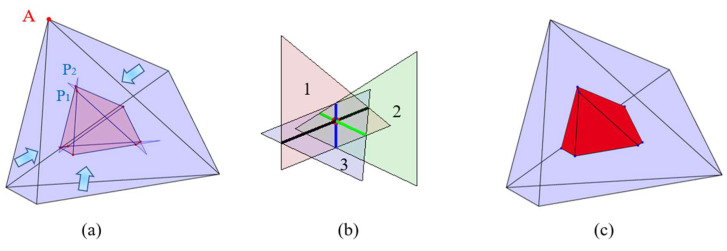
Schematic diagram of (**a**) the uniform thickness shrinkage of each surface of the irregular hexahedral particle, (**b**) the intersection of three faces at a point, and (**c**) the new convex polyhedron after the shrinkage of the irregular hexahedral particle.

Taking the tetrahedron in Figure 5a as an example, when all faces of a regular polyhedron undergo uniform inward shrinkage with an equal thickness at time *t_j_*, the shrinking thicknesses of all faces are equal. The same principle applies to irregular polyhedra. Theoretically, each triangular face of an irregular polyhedron shrinks in an equal proportion along the direction of the inner normal vector of the respective face during this shrinkage process, and it shrinks into a point *O* ultimately, as depicted in Figure 5b. However, since all the dihedral angles of an irregular polyhedron are not equal, and the original shape cannot be contracted in an orderly fashion based on the same dihedral angle as a regular polyhedron does; the small triangular surfaces generated during the shrinkage process of the irregular polyhedron are interspersed and staggered, as illustrated in Figure 6a.

Triangular faces of four original polyhedra sharing point *A* as a common vertex will inevitably generate some new vertices during the process of intersecting, as shown in Figure 6a. Since it represents a shrinkage of equal thickness, the shape of the irregular convex polyhedron investigated in this paper will also be the shape of the closed convex polyhedron after shrinking the polyhedron. Moreover, each vertex of a closed convex polyhedron must be formed by the intersection of at least three faces, as shown in Figure 6b. The first step is counting the number of combinations of three faces among all the faces of the original polyhedron. The second step is calculating the intersection points of the three faces in each combination in turn. Finally, all the vertices of the contracted polyhedron can be selected from the set of intersection points calculated in this part. Assuming that the general equation of each face is *A_i_x + B_i_y + C_i_z +D_i_ =* 0, the formula for the points generated by the intersection of three faces is given in Equation (11).

(11) $$\left[\begin{array}{l} A_{1}B_{1}C_{1}\\ A_{2}B_{2}C_{2}\\ A_{3}B_{3}C_{3} \end{array}\right]\left[\begin{array}{l} x\\ y\\ z \end{array}\right]=\left[\begin{array}{l} -D_{1}\\ -D_{2}\\ -D_{3} \end{array}\right]$$

Based on this principle, the computed points are gathered for subsequent selection. The selection criterion is that the perpendicular distance from each new vertex to the original triangular faces forming it equals the contraction thickness, which is the thickness of the inner product *δ_in_*_,*Deq*_. This process eliminates some points that are located outside the new convex polyhedron, and the final points obtained are the vertices of the contracted polyhedron. By implementing this approach, the shape of the new convex polyhedron can be determined by connecting all the screened vertices and re-triangulating them, as shown in Figure 6c.

**Figure 7:**
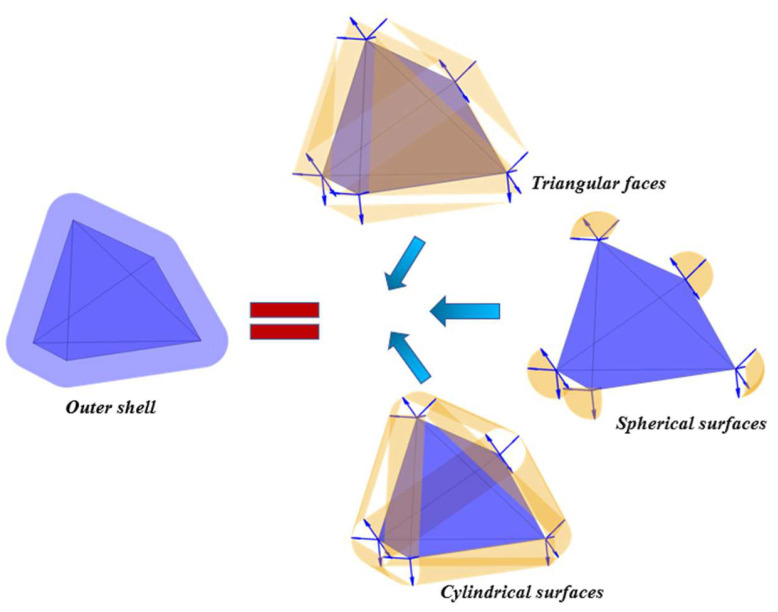
The structure of the outer product layer of the irregular hexahedral particle.

According to previous studies [24], the shell-like outer layer formed through outward uniform-thickness expansion of an irregular polyhedron comprises three distinct geometric components, as shown in Figure 7.

Step 1. Translated planar surfaces.

Step 2. Cylindrical surfaces along edges.

Step 3. Spherical surfaces at vertices.

**Figure 8:**
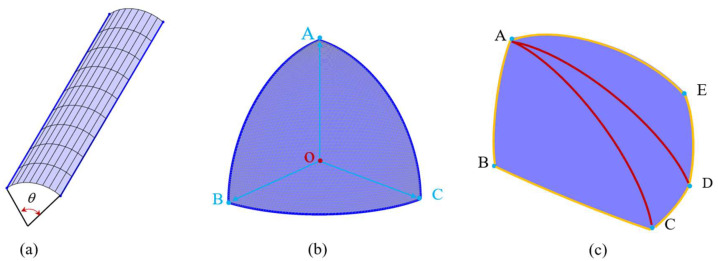
Schematic diagram of (**a**) mesh delineation of cylindrical arc, (**b**) mesh delineation of three spherical arcs with directed radii, and (**c**) triangular sectioning of five spherical arcs with directed radii.

By translating an outside faces of the original polyhedron along the outer normal vector by a thickness *δ_out_*_,*Deq*,*j*_, which denotes the thickness of the outer product layer, a new triangular surface known as the plane is created. This part of the cylindrical surfaces evolve from the edges around the polyhedron, where the thickness of outer product layer *δ_out_*_,*Deq*,*j*_ is equal to the distance between these cylindrical surfaces and the surface of initial polyhedron. It resembles a part of a cylinder. The lateral surface of this section of the cylinder has two generatrixes, and the two generatrixes are obtained by, respectively, translating the edges by a distance of *δ_out_*_,*Deq*,*j*_ along the outer normal vectors of the adjacent planes which this edge is located. The included angle between these two outer normal vectors is *γ*. This angle *γ* is equal to the supplementary angle of the dihedral angle of the two adjacent planes in the original polyhedron where this edge is located. It is worth noting that since the dihedral angles corresponding to all the edges of the convex polyhedron are less than 180°, the angle corresponding to this portion of the cylindrical lateral surface must also be less than 180°. The portion of the cylindrical lateral surface corresponding to each edge is less than half of the entire cylindrical lateral surface. The cylindrical curved surface is shown in Figure 8a.

Moreover, a spherical arc is a set of all points outside the polyhedron whose distance to a certain vertex *O* is equal to the thickness of the outer product layer *δ_out_*_,*Deq*,*j*_. Thus, the entire arc-surface lies within a sphere centered at vertex *O*. The radius of this sphere is the thickness *δ_out_*_,*Deq*,*j*_, of the outer product layer, and it contains at least three oriented radii starting from vertex *O*. The direction vectors of these oriented radii correspond to the outer normal vectors of the faces that share vertex *O* as a common vertex. In this paper, the simplest case of three-oriented radii was taken as an example, as shown in Figure 8b. Their intersections with the sphere were points *A*, *B*, and *C*. The planes formed by two adjacent oriented radii intersected the sphere, and the intersection curves were arcs. These arcs were not only the boundaries of the spherical arc but also the boundaries in the axial direction of the previously mentioned cylindrical arc. Once the coordinates of vertex *O* and the coordinates of three points on the spherical surface after choosing three vertices were well determined, any point on the spherical surface could be linearly represented by these three vectors, *OA*, *OB*, and *OC*. The formula for the linear representation is given by Equation (12).

(12) $$P=(1-u-v)\vec{OA}+u\vec{OB}+v\vec{OC}$$

Let *u* ∈ (0,1), *v* ∈ (0,1), and *P* is a point of spherical surface; and *OA*, *OB*, *OC* are three vectors. The *x*, *y*, and *z* coordinate matrices of all points on the spherical arc were obtained, and the complete spherical arc was drawn by meshing, as shown in Figure 8b. In a simple irregular polyhedral, such as an irregular tetrahedra, each of their vertices is formed by the intersection of three planes. However, for a more complex polyhedron, such as an irregular octahedral particle, irregular dodecahedral particle, and irregular icosahedra particle, there are more than three planes intersect at their vertices. For instance, as shown in Figure 8c, the spherical arc surface was bounded by five vertices *A*, *B*, *C*, *D*, and *E*. It is difficult to directly solve for the positions of all points on the surface. For simplicity, this study initially split an arc with more than three directed radii into triangular spherical arcs.

We selected a vertex *A* and connected it to the non-adjacent vertices *C*, *D*, and *E* in sequence; the spherical arc surface *ABCDE* was divided into three parts, namely *ABC*, *ACD*, and *ADE*. After the division was completed, the triangulation process was carried out for each part in order.

**Figure 9:**
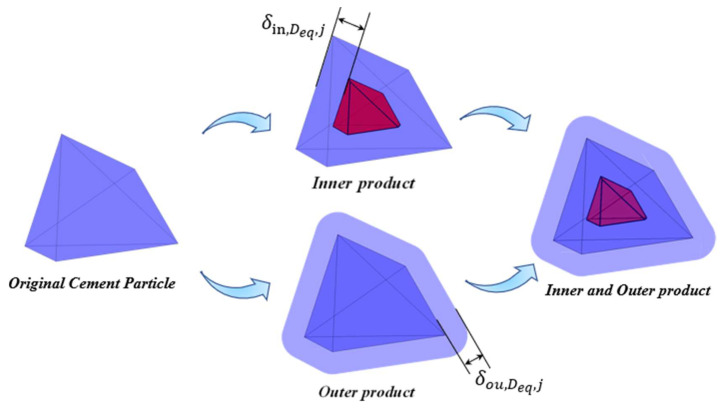
Composition of the hydration products of irregular hexahedral particles.

Based on the above-mentioned construction methods for inner products and outer products, as shown in Figure 9.

**Figure 10:**
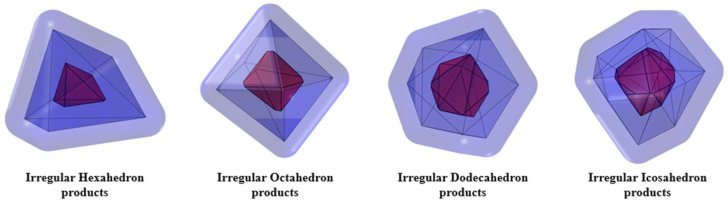
The inner and outer products around the four irregular polyhedral particles.

The shapes of the four polyhedra in Figure 3 after hydration can also be drawn respectively, as shown in Figure 10.

**Figure 11:**
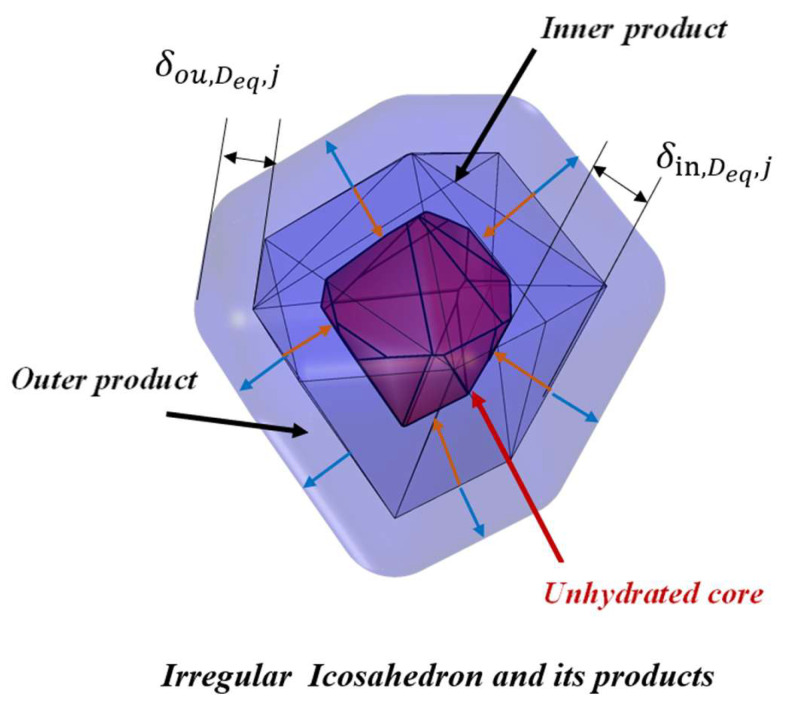
The inner and outer products of an individual irregular icosahedral particle.

Currently, it is unable to account for the variations in shape of each cement particle in the cement paste. Therefore, cement particles having a single irregular polyhedral morphology are briefly studied in this study. An irregular icosahedral particle was employed to simulate cement hydration, and it featured a more intricate composition in its original form, consisting of a combination of vertices, edges, and faces. During the hydration reaction, the inner hydration products were formed in a specific region, which was the part of the cement situated between the original surface and the unhydrated core. Essentially, this region represents the volume of cement that participates in the hydration reaction as a reactant. The shape characteristics of the hydration kinetics of an individual irregular icosahedral particle are shown in Figure 11.

### 3.2. Simulation Mechanism of Hydration of Cement Paste Under Multiple Sized Particle

In this section, the HYD-NSP model in our previous study is adopted to evaluate hydration of irregular polyhedral cement particles. The simulation of cement paste hydration for the particle packing structure of multiple sized cement particles considers the interactions between the external hydration products layer of neighboring particles, including the water absorption effects of embedded particles and capillary pores. Moreover, the permeation rate of cement hydration in different periods is mainly controlled by the mechanism of the two parts: the phase boundary reaction stage, which plays a dominant role in the early stage, and the diffusion reaction stage [25], which dominates in the later stage.

It is notable that with the hydration proceeding, the thickness of the outer product layer of all particles increases rapidly, and small adjacent particles will gradually become embedded in the outer product layer of the large particle, affecting the hydration process and the morphology of the hydration product. Due to the embedded small particles, the thickness of the outer product layer is also increased. Meanwhile, the embedded small particles also undergo the hydration process, which further increases the thickness of the outer product layer of the center particle. The increased thickness of the outer product layer embeds more small cement particles. In time, this process eventually reaches dynamic equilibrium. Let the maximum diameter of the cement particles in the shell be *D_eq_*. The increment of penetration depth increases Δ*δ_in_*_,*Deq*,*j*+1_ in increasing Δ*t_j_* time for particle *D_eq_*. Based on the HYMOSTRUC model, the penetration rate of cement particle hydration over time is given in Equation (13).

(13) $$\frac{\Delta \delta_{in,D_{eq},j+1}}{\Delta t_{j+1}}=\frac{k_{i}\times F_{1}\left(T\right)\times {\left[F_{2}\left(T\right)\right]}^{i}\times \Omega_{1}\times \Omega_{2}\times \Omega_{3}}{{\left[{\left(\delta_{D_{eq},j}\right)}^{i}\right]}^{\beta_{1}}}$$

where *k_i_* is a constant; depending on the hydration mechanism, cement composition, and degree of hydration, the constant *β*_1_ needs to be determined through appropriate experiments. *i* = 0 and 1 represent the two phases of the dominant hydration process, i.e., the phase boundary reaction phase and the diffusion-controlled reaction phase, respectively, and the average value of the reaction rate within the phase boundary reaction phase tends to be *k*_0_ in units of μm/h, as calculated by Equation (14).

(14) $$k_{0}=0.02+6.6\times 10^{-6}\times {\left[C_{3}S\%\right]}^{2}$$

where *C_3_S*% refers to the proportion of tricalcium silicate (*C_3_S*), a clinker mineral phase, in the mass of the cement. The thickness of the hydration products *δ_Deq_*_,*j*_ increases with the progress of cement hydration. The hydration process enters a diffusion-controlled phase when this thickness exceeds the transition thickness *δ_tr_*. In the diffusion-controlled phase, the reaction rate follows the Equation (15).

(15) $$k_{1}=k_{0}\times {\left(\delta_{tr}\right)}^{c}$$

In this model, *c* is taken to be 2 [26]. The temperature function *F*_1_(*T*) represents the impact of the curing temperature on the hydration process in the cement paste, expressed in the form of activation energy. Meanwhile, *F*_2_(*T*) represents the influence of the temperature-induced densification of the diffusion layer on the rate of water penetration when the hydration reaction enters the diffusion phase. The reduction factor Ω_1_ mainly takes into account the dehydration effect. Because the small particles are embedded in the outer product layer of the central particle within each hydration unit, the reduction factor Ω_2_ reflects the situation where the water in the capillary pores cannot be fully utilized during the hydration process. Additionally, as the hydration process progresses, the amount of water in the cement paste gradually decreases. Therefore, a reduction factor Ω_3_ is introduced to quantify the overall effect resulting from the decrease in water volume during the hydration process. Here, *δ_Deq_*_,*j*_ denotes the total thickness of the inner and outer products layer at a specific time *t_j_*.

The above steps can be used to obtain the thickness of the inner products layer and the degree of hydration of cement particles of various particle sizes in the whole cement paste at any time. To calculate the final thickness of the outer layer product *δ_Deq_*_,*j*_ at the end of time *t_j_*, the total volume of the outer product layer of individual cement particle of particle size *D_eq_* under unaffected conditions, *v_out_*_,*Deq*,*j*_, is determined by Equation (16).

(16) $$v_{out,D_{eq},j}=\left(\upsilon -1\right)\times \alpha_{D_{eq},j}\times \frac{\pi D_{eq}^{3}}{6}$$

where *υ* is the volume expansion ratio of the hydration products to the reacting cement at the curing temperature *T* [27], calculated by Equation (17).

(17) $$v\left(T\right)=2.2\times e^{-2.8\times 10^{-5}\times T^{2}}$$

The influence of other small particles within the space unit is taken into account. Therefore, the total volume *v*′*_out_*_,*Deq*,*j*_ of the outer layer product [26] of an actual individual cement particle is given by Equation (18).

(18) $$\begin{array}{c} v\prime_{out,D_{eq},j}=v_{out,D_{eq},j}+v\prime_{out,D_{eq},j}\times \xi_{D_{eq},sh}\times \left[\alpha_{<D_{eq},j}\times \left(\upsilon -1\right)+1\right]\\ =\frac{\alpha_{D_{eq},j}\times \frac{\pi D_{eq}^{3}}{6}\times \left(\upsilon -1\right)}{1-\xi_{D_{eq},sh}\times \left[\alpha_{<D_{eq},j}\times \left(\upsilon -1\right)+1\right]} \end{array}$$

where *ξ_Deq_*_,*sh*_ is the density of cement within the shell of the spatial unit, excluding the largest particle *D_eq_*. *α_<Deq_*_,*j*_ is the total hydration rate of all particles smaller than *D_eq_* at the time *t_j_*; it can be understood as the sum of the degrees of hydration of all particles in the shell smaller in diameter than the center particle *D_eq_* at the time *t_j_* and dividing it by the cement admixture. This is calculated by Equation (19).

(19) $$\alpha_{<D_{eq},j}=\alpha_{\leq D_{eq}-1,j}=\frac{1}{G\left(D_{eq}-1\right)}\sum_{x=D_{eq\_min}}^{D_{eq-1}}\alpha_{x,j}\times g\left(x\right)$$

Based on the Minkowski addition theory [28,29], the outer product can be understood as a shell-like structure. The structure is formed by the original polyhedral particles and spherical particles with a diameter equal to the thickness of the outer product. The volume of such an equal-thickness shell can be given according to the Steiner formula [30,31] in Equation (20).

(20) $$v\prime_{out,D_{eq},j}=S\delta_{out,D_{eq},j}+2\pi B\delta_{out,D_{eq},j}^{2}+4\pi \delta_{out,D_{eq},j}^{3}/3$$

where *S* is the external surface area of the particle, and *B* is the average caliper diameter of the particle [32], which, for polyhedral particles, is calculated by Equation (21).

(21) $$B=\frac{1}{4\pi }\Sigma \alpha_{i}\times l_{i}$$

where *α_i_* is the dihedral angle between two triangular faces sharing the edge in the polyhedron, and *l_i_* is the length of this edge.

Consequently, the thickness of the outer product layer *δ_out_*_,*Deq*,*j*_ can be derived from the outer product layer volume by further solving the cubic equation.

### 3.3. Hydration Simulation Process of Multi-Sized Irregular Polyhedral Cement Paste

Before simulating the hydration process of a cement particle system at multiple particle sizes, the particle packing structure of the cement must be constructed based on the Rosin–Rammler particle size distribution function in Equation (4). This primary work was to randomly fill the fresh cement paste in the RVE with irregular polyhedral particles. This process was accomplished using the random sequential addition procedure [33], and it was necessary to guarantee that there are no overlaps between these particles. The separation axis algorithm [34,35] was adopted to accomplish the overlap detection for irregular icosahedral particles. In addition, this study employed periodic boundary conditions to eliminate the influence brought by boundary impact.

**Figure 12:**
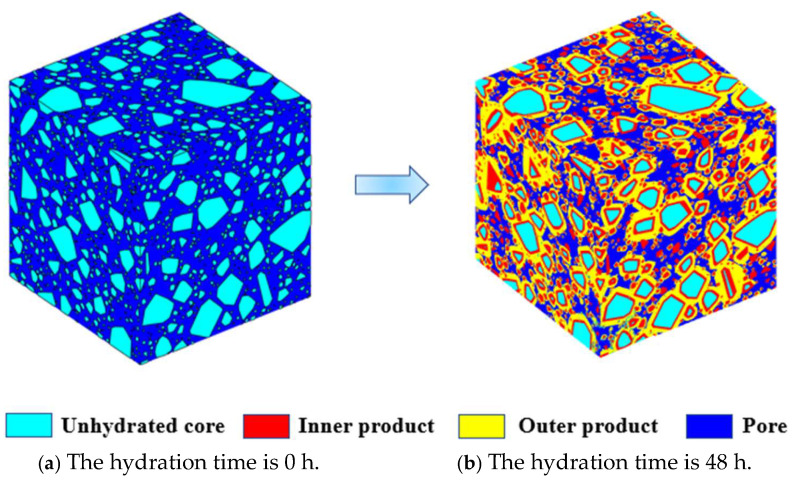
Microstructure of irregular icosahedral granular cement paste before and after hydration.

The schematic illustrations of the overall hydration process in fresh and harden states are presented in Figure 12. More precisely, Figure 12a shows the packing system of irregular icosahedral particles, while Figure 12b visualizes a microstructure of the cement paste 48 h after hydration.

**Figure 13:**
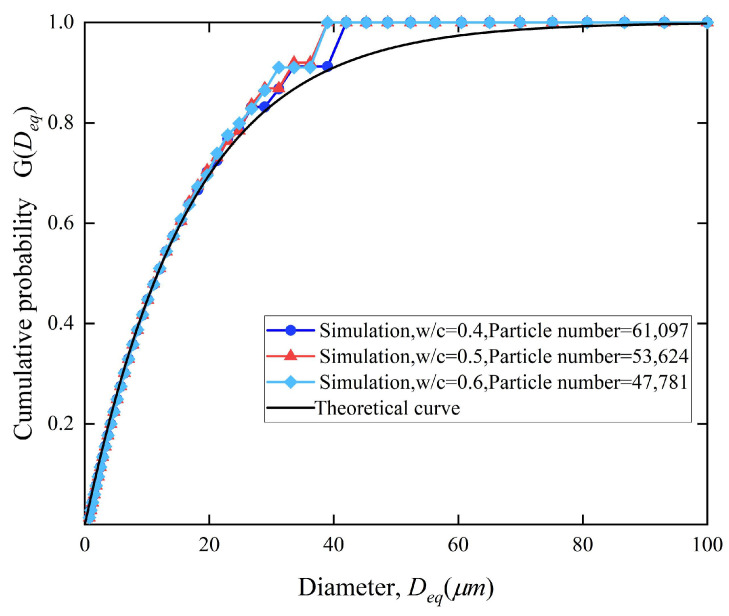
Cumulative probability curves of particle size distribution at three *w*/*c* ratios vs. theoretical curves of Rosin–Rammler distribution.

In the simulation, the parameters were set as follows: the container size was set as 100 × 100 × 100 μm^3^, *w*/*c* = 0.4, *δ_tr_* = 2.6 μm, and *k*_0_ = 0.03 μm/h [7]. To ensure the stable progress of hydration in the cement paste, the fitness of cement particles should be controlled. Therefore, the minimum diameter of the cement particles was set as 0.5 μm, and the maximum diameter was set as 100 μm. It can be seen in Figure 13 that the simulated particle size distribution curves under three different *w*/*c* ratios all showed good fitting degrees with the curves of the Rosin–Rammler theoretical equation. The mass of cement decreased gradually with the decrease of the *w*/*c* ratio, and the number of cement particles increased obviously. The specific chemical composition of cement is shown in Table 2.

**Figure 14:**
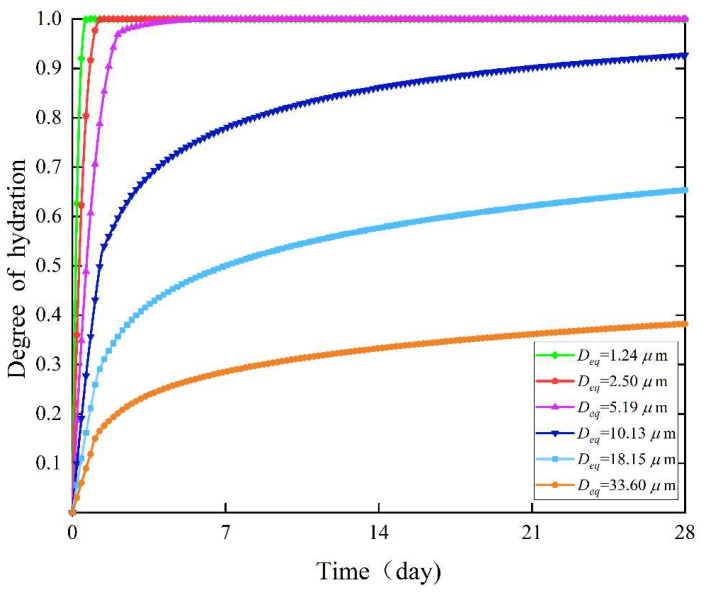
The curves of the degree of hydration changes of irregular icosahedral cement particles with six different diameters.

According to the size distribution of cement in Figure 13, six particles with different diameters were randomly selected to observe the changes in the degree of hydration within the hydration age, as shown in Figure 14.

The degree of hydration curves for irregular icosahedral cement particles with six different diameters (*D_eq_* = 1.24, 2.50, 5.19, 10.13, 18.15, and 33.60 μm) exhibited obvious differences. The overall trend of the degree of hydration decreased with the increase in particle diameter. Moreover, small particles reached complete hydration rapidly in the early stage of hydration, while the reaction rate of large particles gradually slowed down over time.

## 4. Discussion

The veracity of the numerical simulation was confirmed in this section by comparing the simulated data with the experimental data of earlier researchers. Furthermore, the inherent connections among the degree of hydration of the cement paste, the shape of cement particles, *w*/*c* ratio, and the temperature during the hydration process were explored. We also investigated the connection between the shape of cement particle and the porosity of the cement paste.

### 4.1. Effect of Particle Specific Surface Area on the Degree of Hydration

**Figure 15:**
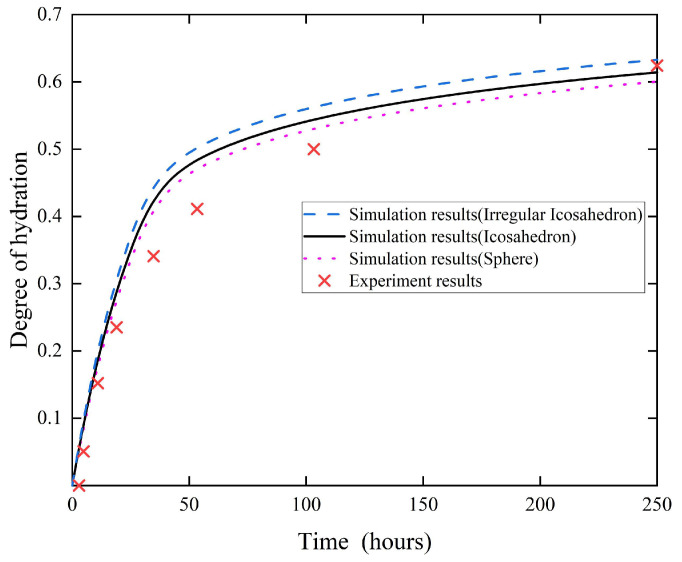
Effect of shape on the degree of hydration, Experiment data are sourced from [36].

It can be observed from Figure 15 that under the same condition (curing temperature *T* = 20 °C and the *w*/*c* = 0.4), the degree of hydration of the three types of particles gradually increased with the increase of hydration time. The growth trends of these three curves indicate that within the initial 48 h, the degree of hydration increased rapidly, while in the subsequent stage, the growth rate experienced a progressive slowdown. This is mainly attributed to the particle size distribution of the cement, which was dominated by small particles. The reason behind this is that in the initial stage, the water was sufficient, and it was easy for water to contact with cement particles. With the increase of water penetration depth, the small particles that constituted the majority were gradually fully hydrated. The distance from the surface to the inner core was relatively long, and the water on the surface was blocked by multiple layers for medium and large-sized particles. Consequently, after a certain period of hydration, water penetration difficulties in large particles caused the hydration rate to gradually decrease. Correspondingly, the increase in the degree of hydration also slowed down gradually. The simulation results were carefully compared with the experimental data of Ye [36] to comprehensively verify the reliability of the numerical simulation.

Meanwhile, it can also be observed from Figure 15 that during the process of cement hydration for the three shapes, the degree of hydration increased rapidly, and the differences between them became more and more obvious. The degree of hydration of the irregular icosahedron was slightly higher than that of the icosahedron, and the degree of hydration of the icosahedron was slightly higher than that of the sphere. Since the hydration model in this paper evolved from HYMOSTRUC model, internal stresses were not taken into account in this simulation. Therefore, the main reason for this is the differences in the specific surface areas of the three. The specific surface area was defined as the surface area per unit mass of an object, and in the cement paste, it was mainly influenced by the shape of the cement particles and the particle size distribution of the cement paste. Taking the above three types of particles as an example, the calculation formula for the specific surface area is given in Equation (22).

(22) $$SSA=\sum_{D_{eq\_min}}^{D_{eq\_max}}\frac{\pi D_{eq}^{2}N(D_{eq})}{s\times m_{c}}$$

**Figure 16:**
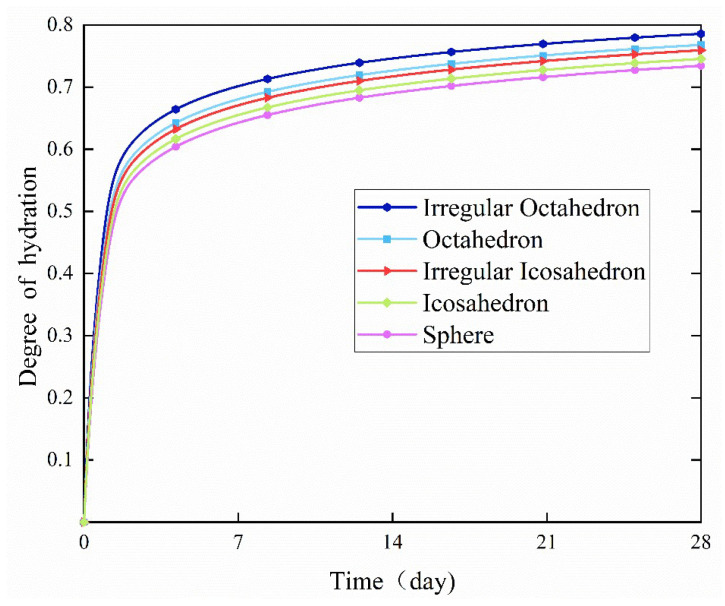
Effect of the numbers of faces on the degree of hydration.

Under the Rosin–Rammler distribution of *w*/*c* = 0.4, the specific surface areas of cement paste containing spherical particles, icosahedral particles, irregular icosahedral particles, octahedral particles, and irregular octahedral particles were 324.15 m^2^/kg, 345.09 m^2^/kg, 372.85 m^2^/kg, 391.59 m^2^/kg, and 418.35 m^2^/kg, respectively. Irregular octahedral particles had the largest specific surface area among these particles. Hence, under the same conditions, due to their largest specific surface area, these irregular octahedral particles had a larger contact area with the water. This could promote water penetration into the cement particles, thus accelerating the hydration reaction. Thus, it can be further deduced that under the same conditions, polyhedral particles with fewer faces and irregular shapes would possess a larger specific surface area; thus, they had a higher degree of hydration reaction, as shown in Figure 16.

In addition, at the subsequent stage of hydration, all small particles were fully hydrated, the growth rate of the degree of hydration slowed down, and the difference in the trends of the hydration levels of the three particles was gradually weakened. This result is consistent with the findings of Bullard et al. [37]; they investigated the effect of spherical particles on the degree of hydration and concluded that the shape of the cement particles had a significant effect on the hydration behavior at the early stages. However, this effect was significantly reduced at the later stages.

### 4.2. Effect of w/c Ratio

**Figure 17:**
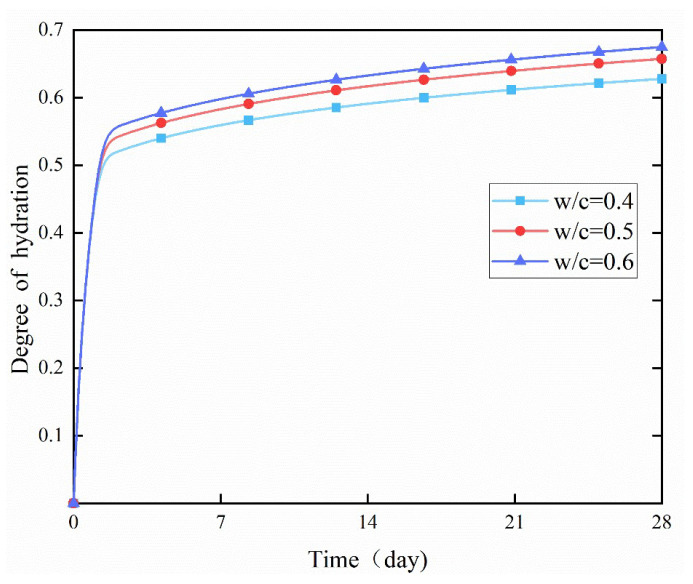
Effect of the *w*/*c* ratio on the degree of hydration at 15 °C.

In addition, the effect of the *w*/*c* ratio on the degree of cement hydration was considered, and *w*/*c* ratios of 0.4, 0.5, and 0.6 were introduced. It can be seen from Figure 17 that the effect of the *w*/*c* ratio on the degree of hydration was negligible during the first 48 h of hydration. However, after this time, the degree of hydration gradually increased as the *w*/*c* ratio increased. This is because a higher *w*/*c* ratio supplies more water for the hydration process, facilitating a higher degree of hydration.

### 4.3. Effect of Curing Temperature

**Figure 18:**
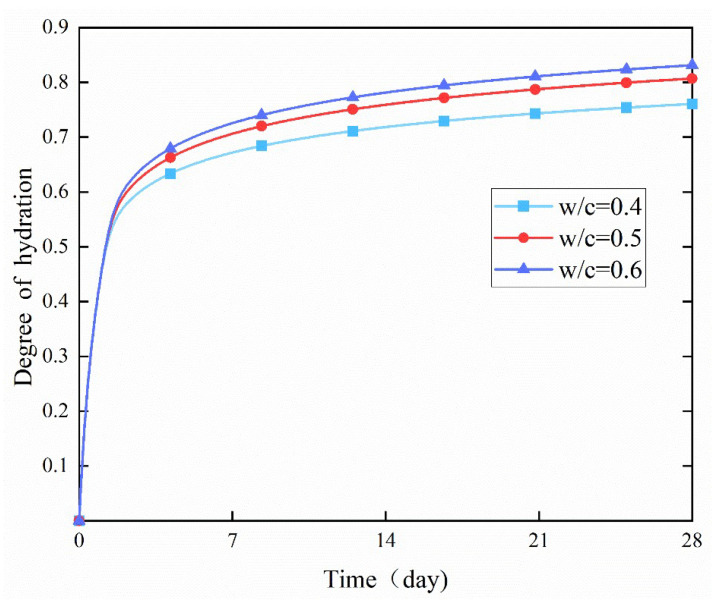
Effect of the *w*/*c* ratio on the degree of hydration at 20 °C.

**Figure 19:**
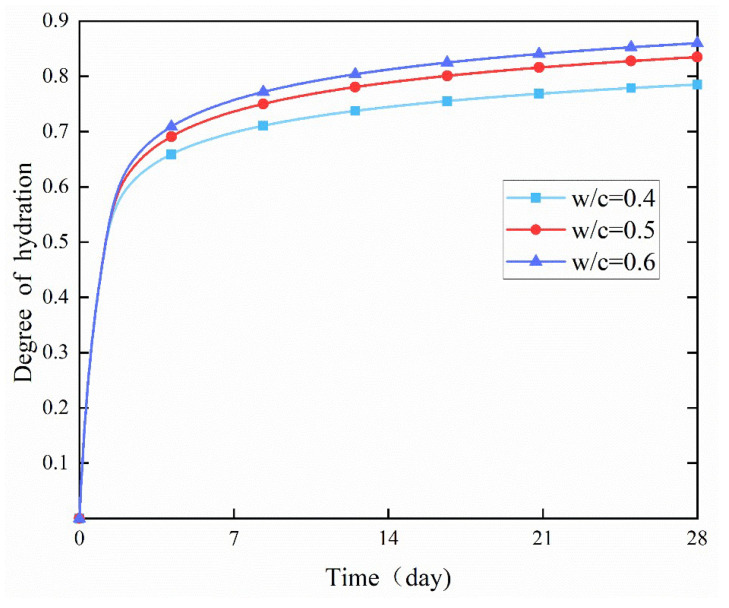
Effect of the *w*/*c* ratio on the degree of hydration at 30 °C.

The hydration rate of cement was significantly accelerated with the increase of the surrounding curing temperature, and the degree of the hydration reaction also increased remarkably, as shown by comparing the degree of hydration of the three curves in Figure 17, Figure 18 and Figure 19 (the change curves of degree of hydration at three temperatures of 15, 20, and 30 °C). Moreover, the increase in the degree of hydration when the temperature rose from 15 °C to 20 °C was more pronounced than that when the temperature rose from 20 °C to 30 °C. It indicates that low temperatures have a stronger inhibitory effect on the cement hydration reaction, suggesting that the optimal hydration temperature for cement is above 20 °C.

### 4.4. Porosity

**Figure 20:**
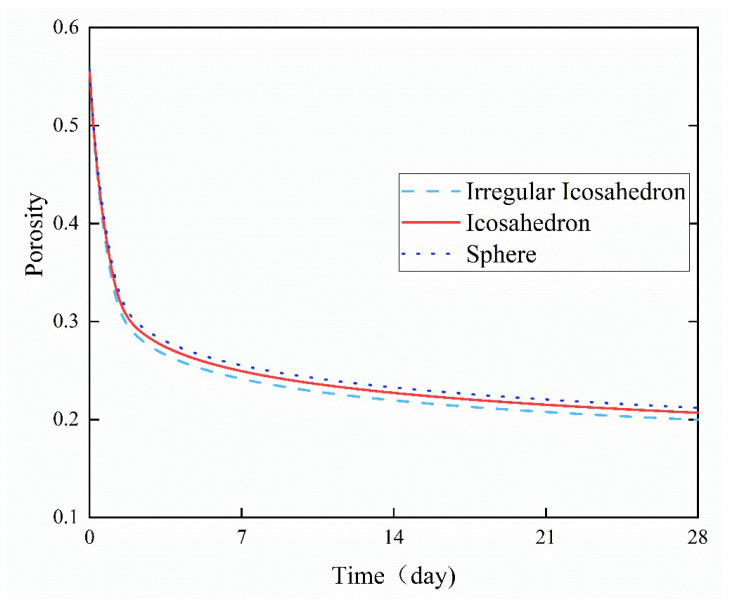
Variation of the porosity of the three shapes of particles at *w*/*c* ratio = 0.4.

Porosity serves as a crucial parameter for comprehensively characterizing the properties of porous media materials, as reported in references [38,39]. It can be observed in Figure 20 that a large number of small particles hydrated rapidly shortly after cement hydration, resulting in a quick consumption of water. The hydrated outer product layer rapidly occupied the positions originally occupied by water in the original structure and filled the pores, so the porosity of the cement paste with the three types of particle shapes decreased drastically shortly after the start of hydration. The porosity is calculated by Equation (23).

(23) $$\varphi_{por}=\frac{V_{por}\left(\alpha_{j}\right)}{V_{RVE}}=\frac{\rho_{c}}{\rho_{w}+\rho_{c}\times w_{c}}\left(w_{c}-0.3375\times \alpha_{j}\right)$$

The hydration rate slowed down significantly, and the rate of porosity reduction also slowed down gradually with the continuous progress of hydration, especially when the basic hydration of small-size cement particles was completed. The irregular icosahedral particles had the largest specific surface area under the same conditions of increasing temperature and *w*/*c* ratio. They exhibited the fastest decline in porosity and the lowest post-hydration porosity in the microstructure, which indicate that they generated the greatest amount of hydration products. This was followed by icosahedral and spherical particles in sequence, suggesting that the specific surface area of cement particles significantly affected the porosity of the hydrated microstructure.

## 5. Conclusions

In this study, a numerical simulation of the continuum hydration process based on irregular polyhedral particles was conducted. In our previous studies, the HYD-NSP model has been applied to a continuum hydration simulation of ideal particles such as spherical and regular polyhedral particles. This model was extended and applied to simulate the hydration of irregular particles. Firstly, the geometric structure of irregular polyhedral particles was constructed. Secondly, the particle packing structure was completed by combining the Rosin–Rammler distribution and the random sequential addition procedure. Thirdly, the continuum hydration model HYD-NSP was employed to simulate the hydration kinetics of particles with multiple particle sizes. Finally, the following conclusions could be drawn:

(1) The degree of hydration grew quickly within 48 h of the start of hydration, and there was no apparent difference in the degree of hydration between the various particle forms. After the first 48 h, the rate of hydration decreased, and changes in the level of hydration under various particle shapes gradually slowed.
(2) The specific surface area of irregular octahedral particles was higher than that of spherical particles, regular octahedral particles, regular icosahedral particles, and irregular icosahedral particles. Results showed that irregular polyhedral particles had a greater specific surface area than regular polyhedral particles when the number of faces was equal. The specific surface area of irregular polyhedral particles increased with the number of faces. Furthermore, the rate of hydration increased with the specific surface area. The primary reason for this is that a higher specific surface area improves interaction with water, which makes it easier for water to enter the particles’ core and accelerates the hydration reaction.
(3) In the early stage, different water-to-cement (*w*/*c*) ratios had no obvious effect on the degree of hydration. However, in the later stage, the degree of hydration increased significantly with the increase of the *w*/*c* ratio. This was because a higher *w*/*c* ratio allowed more water to react with cement particles per unit volume, thereby increasing the hydration rate.
(4) An increase in the curing temperature of the surrounding environment also promoted the acceleration of the hydration rate of cement, while low temperatures had a more obvious inhibitory effect on the hydration reaction. When the curing temperature rose to 20 °C, the growth rate of the early-stage hydration reaction slowed down.
(5) Among the three particle shapes, irregular icosahedral particles had the highest specific surface area, and their porosity decreased the fastest, followed by icosahedral particles and spherical particles. The porosity of the cement paste decreased rapidly in a short period of time after hydration began, but at a slower rate as hydration proceeded.

## Figures and Tables

**Table 1:** Intervals range of particle diameters under Rosin–Rammler distribution.

Interval Sequence Number	Diameter	Interval Range
0	*D_eq_*_,0_ (*D_eq_min_*)	[*D_eq_*_,0_, *D_eq_*_,1_]
1	*D_eq_*_,1_	[*D_eq_*_,1_, *D_eq_*_,2_]
…	…	…
*i*	*D_eq_*_,*i*_	[*D_eq_*_,*i*−1_, *D_eq_*_,*i*_]
…	…	…
*M*	*D_eq_*_,*M*_ (*D_eq_max_*)	[*D_eq_*_,*M*−1_, *D_eq_*_,*M*_]

**Table 2:** Constituents of cement [36].

Phases	Weight (%)
C_3_S	63
C_2_S	13
C_3_A	8
C_4_AF	9

## Data Availability

The original contributions presented in this study are included in the article. Further inquiries can be directed to the corresponding author.
